# Analysis of compliance and efficacy of integrated management of whole process in the choice of percutaneous full-endoscopic surgery for patients with cervical disc herniation

**DOI:** 10.1186/s13018-020-01920-2

**Published:** 2020-09-04

**Authors:** Zhongyan Jiang, Ansu Wang, Chong Wang, Weijun Kong

**Affiliations:** 1 Department of Orthopedics Surgery, Affiliated Hospital of Zunyi Medical University, No.149 Dalian Road, Zunyi, 563000 Guizhou China; 2 Department of Orthopedics Surgery, The Second Affiliated Hospital of Zunyi Medical University, No.1 Xinpu Road, Zunyi, 563000 Guizhou China

**Keywords:** Cervical disc herniation, Integrated management mode, Whole course, Percutaneous endoscopy, Discectomy, Compliance

## Abstract

**Background:**

Percutaneous spinal endoscopy is a new type of surgery for the treatment of cervical disc herniation. It can avoid the complications of the classic anterior cervical discectomy and fusion (ACDF) approach and the risk of adjacent spondylosis. How can we effectively improve patients’ awareness of spinal endoscopy and their election of endoscopic techniques?

**Objective:**

To analyze the compliance and clinical effect of the integrated management of the whole process in the choice of percutaneous full-endoscopic surgery for patients with cervical disc herniation.

**Methods:**

Retrospective analysis of 72 patients with cervical disc herniation undergoing surgery in our hospital from August 2015–August 2017 was performed. The whole-process integrated management model was used for all the patients. The 36 patients in the experimental group were treated by percutaneous full-endoscopic cervical discectomy, and the 36 patients in the control group were treated by ACDF. The postoperative feeding time, time to get out of bed, length of hospital stay, compliance, clinical efficacy, and recurrence rate of neck pain were observed. Changes between the preoperative and postoperative pain visual analog scale (VAS) scores and neurological function Japan Orthopaedic Association (JOA) scores were assessed.

**Results:**

The postoperative feeding time in the experimental group was 8.319 ± 1.374 h, the postoperative time to get out of bed was 16.64 ± 3.728 h, and the hospitalization time was 6.403 ± 0.735 days. The excellent and good clinical efficacy rate was 91.67%, the compliance rate was 88.89%, and the neck pain recurrence rate was 5.56%. The postoperative feeding time in the control group was 26.56 ± 9.512 h, the postoperative time to get out of bed was 45.06 ± 9.027 h, and the length of hospital stay was 8.208 ± 0.865 days. The excellent and good clinical efficacy rate was 88.89%, the compliance rate was 69.4%, and the neck pain recurrence rate was 8.33%. There was no significant difference between the two groups in the excellent efficacy rate and the neck pain recurrence rate, *p* > 0.05. The compliance rate in the experimental group was better than that in the control group, and the difference was statistically significant, *p* < 0.05. The hospitalization time of the experimental group was significantly lower than that of the control group, and the difference was statistically significant, *p* < 0.05. The postoperative VAS scores and JOA scores of the two groups were significantly better than the preoperative scores, and the difference was statistically significant, *p* < 0.05; there was no significant difference between the two groups, *p* > 0.05.

**Conclusion:**

The integrated management of the whole course can effectively improve the compliance of patients with cervical disc herniation receiving endoscopic treatment, yield the same treatment effect as the classic operation, shorten the hospitalization time, speed up the turnover of hospital beds, and improve satisfaction with medical quality and is worthy of clinical application.

Cervical disc herniation (CDH) is a group of diseases based on cervical disc degeneration. The spinal cord and nerve roots are compressed by the protruding intervertebral disc due to a slight external force or no definite inducement, which causes neck and shoulder pain with upper limb radiation pain as the main clinical symptoms, or a small proportion of patients with limb sensory disorders, decreased muscle strength, and other symptoms of spinal cord compression [1]. Symptoms can be relieved in most patients by conservative treatment, but some patients still need surgical treatment to relieve symptoms. The unhealthy postoperative living or work habits of some patients result in the recurrence of symptoms and place a substantial economic burden on the patient [2]. Therefore, maintenance of postoperative efficacy and prevention of the recurrence of symptoms are the focus of clinical treatment and follow-up [3]. Anterior cervical discectomy and fusion (ACDF) is the standard surgical procedure for the treatment of CDH, but may cause postoperative complications such as swallowing discomfort, foreign body sensation in the pharynx, and degeneration of adjacent cervical segments [4]. To reduce the incidence of surgical trauma and related iatrogenic complications, minimally invasive percutaneous endoscopic techniques have gradually been developed for spinal surgery. Spinal full-endoscopic techniques appear to be more advanced than ACDF due to clear visualization, minimal trauma, accurate resection of the herniated lesions, and the cosmetic appearance of the incisions [5–7]. According to the position of the herniated disc, we utilized either a percutaneous anterior or posterior approach for the full-endoscopic resection of herniated intervertebral discs. The posterior percutaneous endoscopic technique can be used only for posterolateral or foraminal disc herniation based on spinal anatomy [8, 9]. In terms of the surgical approach for central or paracentral CDH, we performed an anterior percutaneous transcorporeal full-endoscopic nucleus pulpotomy of the cervical disc to maximally preserve the function of the cervical spine [10, 11]. To investigate the effect of the integrated management of the whole course model on the compliance of and curative effect in CDH patients undergoing full-endoscopic treatment, this study retrospectively analyzed 72 CDH patients undergoing surgical treatment in our hospital in terms of the treatment efficacy, compliance, and recurrence rate of neck pain.

## Information and methods

### Inclusion criteria

(1) For patients with single-segment CDH, the symptoms and signs of the patients were consistent with the MRI examination; (2) the patient received systematic conservative treatment for more than 1 month, and the patient’s symptoms did not improve significantly or consistently; and (3) patients volunteered to participate in clinical observation, made an informed choice regarding the surgical plan, and were willing to cooperate with the clinical follow-up.

### Exclusion criteria

(1) Previous history of cervical spine surgery; (2) cervical deformity; (3) severe osteoporosis; (4) complicated with cervical tumor, tuberculosis, or infectious disease; (5) coagulation dysfunction; (6) goiter and cervical lymph node hyperplasia; (7) neuropsychiatric disorders; or (8) other diseases not conducive to surgery.

### General data

A total of 72 patients with CDH who underwent surgery in our department from August 2015 to August 2017 were analyzed. Experimental group (36 cases): percutaneous full-endoscopic technique treatment; control group (36 cases): ACDF treatment. General information on the two groups of patients is shown in Table 1, and there was no statistically significant difference between the groups, *P* > 0.05.

**Table 1:** General patient data

	Number	Gender		Age (Y)	Course (M)	Education level	
		Man	Woman			High school or above	Junior high school or below
EG	36	23	13	59.89 ± 9.077	6.806 ± 3.8641	32	4
CG	36	21	15	61.14 ± 9.859	7.903 ± 4.332	28	8
***t***		*Z* = − 0.512		0.5358	1.235	*Z* = − 1.500	
***p***		0.608		0.5955	0.2250	0.134	

*EP* experimental group, *CG* control group, *Y* years, *M* months

### Methods

**Fig. 1:**
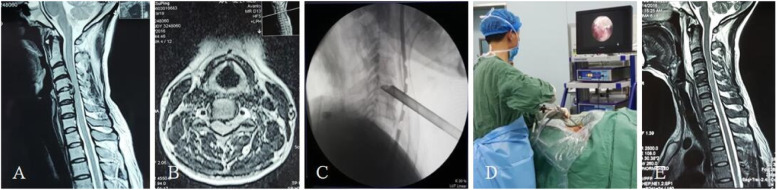
Percutaneous anterior full-endoscopic transcorporeal discectomy and spinal cord decompression. **a** The MRI sagittal image of the cervical spine showed C4/5 cervical disc herniation with local spinal stenosis and spinal cord compression. **b** The MRI axial image of the cervical spine showed C4/5 para-central cervical disc herniation. **c** Intraoperative radiography. **d** Intraoperative view. **e** Postoperative review of cervical spine MRI sagittal image showed complete removal of the C4/5 herniated disc tissue and complete decompression of spinal cord

**Fig. 2:**
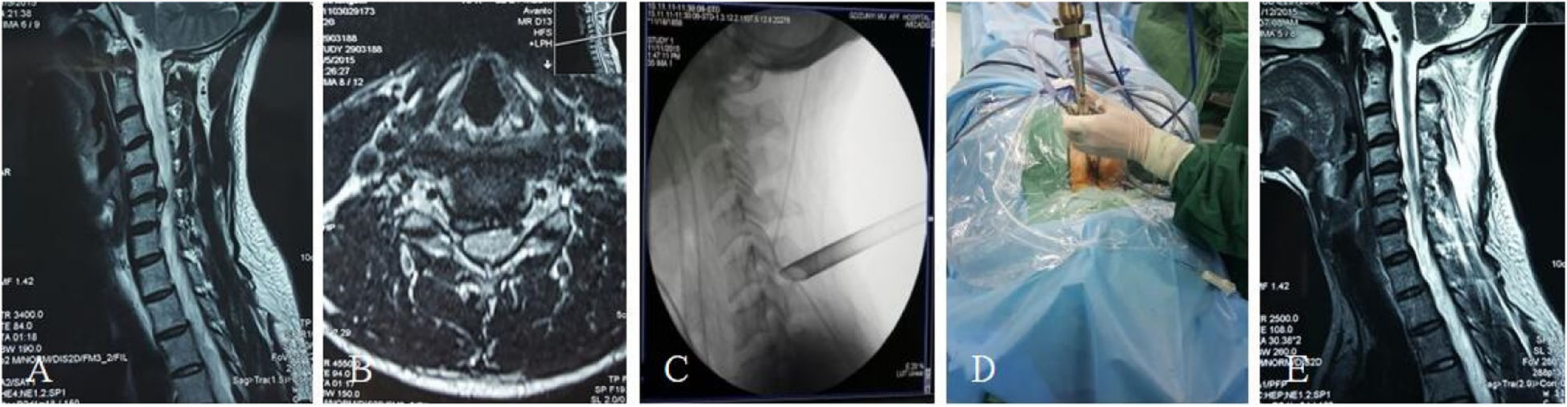
Percutaneous posterior full-endoscopic discectomy and nerve root decompression. **a** The MRI sagittal image of the cervical spine showed C5/6 cervical disc herniation with local spinal stenosis. **b** The MRI axial image of the cervical spine showed C5/6 posterolateral cervical disc herniation with spinal nerve compression. **c** Intraoperative radiography. **d** Intraoperative view. **e** Postoperative review of cervical spine MRI sagittal image showed complete removal of the C5/6 herniated disc tissue and complete decompression of spinal nerve

**Fig. 3:**
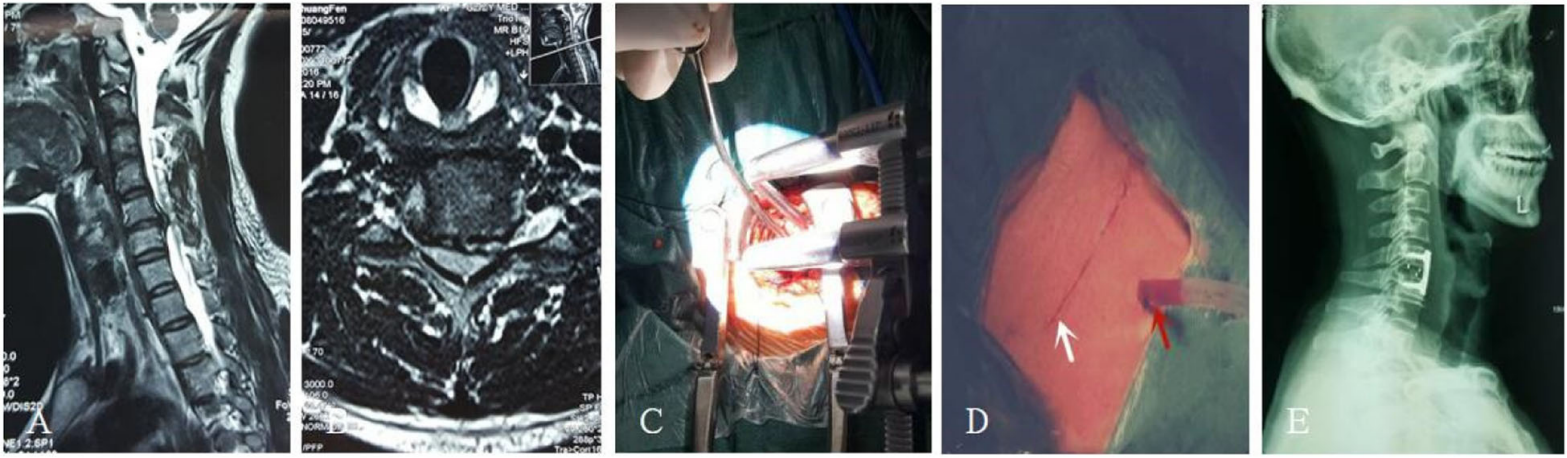
Anterior cervical discectomy and fusion. **a** The MRI sagittal image of the cervical spine showed C5/6 cervical disc herniation with local spinal stenosis and spinal cord compression. **b** The MRI axial image of the cervical spine showed C5/6 para-central cervical disc herniation. **c** Intraoperative microscopic view of cervical discectomy. **d** Postoperative incision diagram. The white arrow indicates the incision, and the red arrow indicates the drainage tube. **e** Postoperative X-ray review showed that the position of intervertebral fusion cage and plate were in good position

After the patient was admitted to the hospital, the resident physician completed medical history collection, a physical examination, medical record writing, and related laboratory and imaging and other auxiliary examinations, and the nurse completed the relevant nursing evaluation and issued the informed notification of hospitalization. A team for the integrated management of the whole course was established: under the guidance of the head of the department, joint ward rounding was carried out by the attending doctor and responsible nurse, and an integrated post-discharge follow-up system was implemented [12, 13]. The executive team implements CDH fixed bed management and provides basic information about the disease, including onset factors, treatment methods, surgical methods, treatment procedures, daily habits, and prevention and health care methods, according to the patient’s cognition [14, 15]. The focus is to make patients aware that spinal minimally invasive technology is the inevitable development direction of contemporary surgical technology. It is an inevitable product of new developments in science and technology, such as channel technology, optical technology, visual imaging technology, and surgical technology. The approach is a new surgical method to maintain the physiological health of patients to the greatest extent. Patients are helped to understand that compared with the classic ACDF operation method, the percutaneous full-endoscopic technique has the advantages of less trauma, faster recovery, no internal plant fixation, lower cost, and better retention of cervical biomechanical stability, which promotes patient rehabilitation confidence [16–18]. At the same time, patients should also be informed of recurrence, spinal cord injury, and other surgical risks. The integrated model involves evaluating the risks of surgery and anesthesia before surgery, eliminating the contraindications for surgery, implementing the three-level physician rounding system and preoperative discussion system, fully communicating with patients and their families, explaining the pros and cons of the two surgical options, and determining the surgical plan based on the patient’s informed understanding and willingness. Endotracheal intubation was used in all patients under general anesthesia. Patients with anterior percutaneous transcorporeal endoscopic decompression were required to have an indwelling gastric tube and retain the imaging agent in the gastric tube to observe the position of esophagus. In the experimental group, the indications of anterior transcorporeal full-endoscopic decompression techniques are central and para-central cervical disc herniation. The indications for the application of posterior percutaneous full-endoscopic decompression techniques are posterolateral and the intervertebral foramen cervical disc herniation. The surgical protocol included percutaneous anterior full-endoscopic transcorporeal discectomy and spinal cord decompression (Fig. 1) or posterior percutaneous full-endoscopic discectomy and nerve root decompression (Fig. 2). In the control group, cervical discectomy, spinal cord decompression, interbody fusion, and internal fixation were performed (Fig. 3). After the operation, the patient’s symptom improvement, normal eating situation, time to get out of bed, and hospitalization time were closely observed, and the patient was scientifically taught how to get out of bed with a cervical collar. On the morning of discharge, the attending doctor and the responsible nurse explained the precautions for discharge to the patient, emphasizing that postoperative recovery for CDH is a long-term process and that it is necessary to develop good living and working habits and a follow-up plan for discharge health education. Patients with poor compliance should be paid more attentions, and their family members should be required to actively monitor them and cooperate [13, 15]. At 3, 6, 12, and 24 months after discharge, the patients were followed up by telephone or regularly reviewed in outpatient clinics to observe their health status, compliance with functional exercise, and evaluation of the clinical effect.

### Observation indicators

The postoperative food intake time, postoperative getting out of bed time, hospitalization time, patient compliance, clinical efficacy, and neck pain recurrence rate were observed, and the visual analog scale (VAS) score of pain and Japan Orthopaedic Association (JOA) treatment score were compared between the two groups preoperatively and postoperatively. Evaluation of efficacy: The JOA score was used to evaluate the clinical treatment effect [19]. The calculation formula of the JOA improvement rate is as follows: postoperative score − preoperative score / (17 − preoperative score) × 100%. A JOA score improvement rate of more than 75% is excellent, 50 to 74% is good, 25 to 49% is acceptable, and less than 25% is poor.

### Statistical methods

Using SPSS 22.0 statistical software, measurement data were expressed as the mean ± standard deviation **(**$\overline{x}$**±s)**, and comparisons between groups were performed by independent samples t tests; count data were tested by the X^2^ test, and *P* < 0.05 was considered statistically significant.

## Results

### Time to eating, time to getting out of bed, hospitalization duration, compliance, and clinical efficacy

All the patients were successfully operated, and their pain symptoms and nerve function were significantly improved after the operation. The time to eating, time to getting out of bed, and hospitalization duration in the experimental group were significantly lower than those in the control group (*P* < 0.05) (see Table 2). There was no significant difference in the rate of excellent and good treatment or the recurrence of neck pain between the two groups (*P* > 0.05), and the compliance rate in the experimental group was better than that in the control group (*P* < 0.05) (see Table 3).

**Table 2:** Comparison of the time to eating, time to getting out of bed and hospitalization duration ($\overline{x}$ ± s, *n* = 36)

	Eating time after surgery (H)	Getting out of bed after surgery (H)	Duration of stay (D)
EG	8.319 ± 1.374	16.64 ± 3.728	6.403 ± 0.735
CG	26.56 ± 9.512	45.06 ± 9.027	8.208 ± 0.865
*t*	11.02	17.34	10.59
*P*	< 0.0001	< 0.0001	< 0.0001

*EG* experimental group, *CG* control group, *H* hours, *D* days

**Table 3:** Comparison of the excellent treatment rate, compliance rate, and neck pain recurrence rate ($\overline{x}$ ± *s*, *n* = 36)

	Excellent treatment rate (%)	Compliance rate (%)	Neck pain recurrence rate (%)
EG	91.67(33/36)	88.89(32/36)	5.56(2/36)
CG	88.89(32/36)	69.4(25/36)	8.33(3/36)
*z*	− 0.397	− 2.291	− 1.025
*P*	0.691	0.022	0.306

*EG* experimental group, *CG* control group

### VAS and JOA score

After the operation, the symptoms of neck pain, upper limb radiation pain, and spinal cord compression were significantly improved, and there was no significant difference between the two groups at any time point, *P* > 0.05 (see Table 4). The postoperative neurological symptoms were significantly improved and returned to normal, and there was no significant difference in the JOA scores at any time point between the groups, *P* > 0.05 (see Table 5). The JOA improvement rates of the groups were excellent, at greater than 75%. There was no significant difference between the groups, *P* > 0.05 (see Table 5).

**Table 4:** Preoperative and postoperative VAS scores ($\overline{x}$ ± *s*, *n* = 36)

	Preoperative	1 week postoperative	6 months postoperative	24 months after surgery
EG	6.264 ± 0.6812	1.319 ± 0.5994^a^	0.5694 ± 0.4653^b^	0.4861 ± 0.4223^c^
CG	6.306 ± 0.7298	1.125 ± 0.8051^a^	0.6806 ± 0.5231^b^	0.6250 ± 0.4532^c^
*t*	0.2504	1.162	0.9523	1.345
*p*	0.8030	0.2491	0.3442	0.1829

There was no significant difference between the groups, *P* > 0.05

*EG* experimental group, *CG* control group

^a^Compared with preoperative, *P* < 0.05

^b^Compared with 1week postoperative, *P* < 0.05

^c^Compared with 6 months postoperative, *P* > 0.05

**Table 5:** Preoperative and postoperative JOA scores ($\overline{x}$ ± s, *n* = 36)

	Preoperative	1 week postoperative	6 months postoperative	24 months postoperative	JOA improvement rate (%)
EG	8.417 ± 0.9599	15.36 ± 1.217^a^	16.24 ± 0.7120^b^	16.44 ± 0.6412^c^	93.54
CG	8.345 ± 0.9321	15.24 ± 1.137^a^	16.33 ± 0.7071^b^	16.39 ± 0.5492^c^	92.82
*t*	0.3114	0.4504	0.5813	0.3948	0.4465
*p*	0.7564	0.6538	0.5629	0.6942	0.6566

There was no significant difference between the groups, *P* > 0.05

*EG* experimental group, *CG* control group

^a^Compared with preoperative, *P* < 0.05

^b^Compared with 1week postoperative, *P* < 0.05

^c^Compared with 6 months postoperative, *P* > 0.05

## Discussion

In the era of rapid information development, people are dependent on computers and mobile phones for life, entertainment, and work. Currently, the onset of CDH is seriously increasing in the younger population. Changes in the morphological structure of the cervical spine are closely related to body posture in daily life and work. Head, neck, and shoulder activities directly affect the physiological function and morphological degeneration of each cervical segment [20, 21]. Clinical studies have shown that the incidence of CDH is closely related to the internal and external imbalance of the cervical spine. Therefore, the restoration of the dynamic and static balance of the cervical spine is the key to effective treatment and prevention of CDH [22]. During hospitalization and after discharge, guided interventions involving the patient’s functional exercise of the cervical spine can help to achieve significant clinical effectiveness [21, 23]. After discharge, the patient’s compliance decreases or disappears, and the early relief of symptoms can cause the patient to forget that good work and life habits are the basis for maintaining the normal function of the cervical spine [24]. Some patients gradually neglect good behavioral habits and cervical functional exercise, leading to the recurrence of disease symptoms or adjacent segment disc herniation. This study targeted patients with CDH and used whole course integrated management of follow-up evaluation guidance to achieve an ideal clinical management effect.

The conventional nursing management mode leads to information asymmetry in medical care, doctor-patient care, and nurse-patient care, and there are several contradictions and inconsistencies [25]. This causes the patient to distrust the doctor and the nurse and even not cooperate with the treatment. The whole course integrated management mode is a new type of treatment and rehabilitation management mode that breaks with the traditional nursing mode [12, 15]. This new model departs from the traditional conservative “subordination model” of medical care, doctor-patient care, and nurse-patient care. Management teams are formed by the doctors in charge and responsible nurses, who participate in the whole process of patients’ recovery and nursing during the perioperative period and after discharge [13–15]. As a result, many hospitals have developed new nursing modes, namely, the continuous treatment mode, the family doctor management mode, and the participation mode, using modern information platforms (such as QQ groups and WeChat groups) to form a new system of seamless docking and three-dimensional integration among doctors, nurses, and patients [26, 27]. The classic procedure for CDH is cervical discectomy, bone graft fusion, and internal fixation. However, long-term clinical follow-up has revealed concerns related to the complications associated with this approach and the acceleration of degeneration of the adjacent segments, in addition to the reappearance of related symptoms and signs of cervical spondylosis [5–7]. Full-endoscopic spinal surgery not only avoids the deficiency of open surgery but also fully preserves the biomechanical stability of the spine [28, 29]. However, patients and nonspecialists have little knowledge of modern spinal full-endoscopic surgery techniques, and they are reluctant to undertake full-endoscopic treatment at an early stage. For patients with CDH who need surgical treatment, because local disc herniation results in limited compression of the spinal cord, percutaneous full-endoscopic surgery not only achieves the purpose of percutaneous targeted removal of disc tissue and decompression of spinal cord but also has a better cosmetic effect, reduces the complications associated with the open surgical approach, better maintains the biomechanical stability of the spine, and avoids the acceleration of degeneration in adjacent segments [30, 31]. Through the integrated management model, doctors, nurses, and patients have a consistent understanding of the percutaneous spinal full-endoscopic procedure, especially because of the physician’s explanation of the etiology, mechanism, operation, spinal cord nerve function recovery, and cervical biomechanical stability. Through this, the responsible nurses fully understand and can cooperate with the doctor’s orders and actively carry out health education and psychological care for the patients. This alleviates patients’ worry and fear because they recognize, understand, and voluntary choose percutaneous full-endoscopic treatment and have increased confidence.

The results showed that there was no significant difference between the two groups, *p* > 0.05, indicating that the newly developed percutaneous spinal full-endoscopic surgery was effective and feasible. The compliance rate in the experimental group was better than that in the control group. The postoperative VAS and JOA scores were significantly better than the preoperative scores (*p* < 0.05). There was no statistically significant difference in the JOA and VAS scores between the two groups, *p* > 0.05. An integrated management model of the whole course enables CDH patients to receive percutaneous endoscopic treatment and obtain effects consistent with the classical surgical procedure while avoiding or significantly reducing the complications associated with that approach; it also improves the patient compliance rate, significantly reduces the length of hospital stay, and reduces the recurrence rate of symptoms [32–34]. Through the integrated management of the whole course, we can promote the consistency of medical and health education, improve the compliance of CDH patients so that they actively cooperate with the treatment, cultivate and maintain good living and work habits, and strengthen awareness of maintaining the health of the cervical spine [3, 14, 24]. The establishment of this new health education model of medical cooperation strengthens the sense of responsibility, makes the personnel in each management group be invested and actively participate, ensures the safety of medical treatment and nursing, avoids the occurrence of medical errors and accidents, and improves the satisfaction of clinicians and patients with nursing work [25, 26]. It also promotes the development of new medical technology, effectively reduces the average length of hospital stay and speeds up the turnover rate of hospital beds. Moreover, it is beneficial for actively carrying out the training and examination of medical staff, encouraging doctors and nurses to stay up-to-date on the frontier of knowledge in their specialty, achieving common progress, and enhancing the building of subject competence [25, 32].

The whole-process integrated management model, as a new type of clinical medical cooperation mode, has achieved good clinical results in a certain professional environment. It is restricted by the degree of cooperation and harmony between medical and nursing staff, the training and assessment of medical and nursing staff, the understanding of leadership, and the degree of support [32, 34]. Its wide application in the clinic still needs to be explored. In addition, considering the rehabilitation needs of different diseases, for some special patients, the whole-process integrated management model can be extended to cooperation with dietitians, rehabilitators, pharmacists, and other multi-professional personnel to discuss and optimize the management plan for the rehabilitation of patients during hospitalization and after discharge [33–35].

## Conclusions

In summary, the implementation of an integrated management model for CDH patients has promoted collaboration and cooperation among physicians, nurses, and patients; improved the compliance of patients with endoscopic treatment and rehabilitation exercises; and yielded the same therapeutic effect as classic surgery. This program is conducive to shortening the length of hospital stay, speeding up the turnover of hospital beds, and improving satisfaction with medical quality control, making it worthy of clinical application.

## Data Availability

The data generated or analyzed during this study are included in this published article [and its supplementary information files].

## Abbreviations

ACDF: Anterior cervical discectomy and fusion
CDH: Cervical disc herniation
JOA: Japanese Orthopaedic Association
VAS: Visual analog scale
